# Peach Kernel Oil Downregulates Expression of Tissue Factor and Reduces Atherosclerosis in ApoE knockout Mice

**DOI:** 10.3390/ijms20020405

**Published:** 2019-01-18

**Authors:** Erwei Hao, Guofeng Pang, Zhengcai Du, Yu-Heng Lai, Jung-Ren Chen, Jinling Xie, Kai Zhou, Xiaotao Hou, Chung-Der Hsiao, Jiagang Deng

**Affiliations:** 1 Guangxi Key Laboratory of Efficacy Study on Chinese Materia Medica, Guangxi University of Chinese Medicine, Nanning 530200, Guangxi, China; ewhao@163.com (E.H.); pangguof@126.com (G.P.); duzhengcai8@163.com (Z.D.); 13257716536@163.com (J.X.); zhoukai46@126.com (K.Z.); 2 Guangxi Collaborative Innovation Center for Research on Functional Ingredients of Agricultural Residues, Guangxi University of Chinese Medicine, Nanning 530200, Guangxi, China; 3 Department of Chemistry, Chinese Culture University, Taipei 11114, Taiwan; lyh21@ulive.pccu.edu.tw; 4 Department of Biological Science & Technology College of Medicine, I-Shou University, Kaohsiung 82445, Taiwan; jrchen@isu.edu.tw; 5 Department of Bioscience Technology, Chung Yuan Christian University, Chung-Li 32023, Taiwan

**Keywords:** peach kernel oil, tissue factor, atherosclerosis, tumor necrosis factor-α, human umbilical vein endothelial cells

## Abstract

Atherosclerosis is the pathological process in arteries due to the plaque formation that is responsible for several diseases like heart disease, stroke and peripheral arterial disease. In this study, we performed in vitro and in vivo assays to evaluate the potential anti-atherosclerosis activity of peach kernel oil. For the in vitro assay, we incubated human umbilical vein endothelial cells (HUVEC) with tumor necrosis factor-α (TNF-α) to induce tissue factors (TF, an essential mediator of hemostasis and trigger of thrombosis) elevation. We found that TNF-α-induced TF elevation was suppressed by peach kernel oil in a dose-dependent manner at both mRNA and protein levels. Peach kernel oil can significantly improve HUVEC viability, protect the endothelial cells, which achieved the goal of prevention of thrombotic diseases. For the in vivo assay, we investigated the effect and mechanism of peach kernel oil on preventing atherosclerotic lesion formation in ApoE knockout mice. Results show that peach kernel oil could reduce total cholesterol, triglyceride, low-density lipoprotein cholesterol levels, elevate the high-density lipoprotein cholesterol level in serum, and reduce the area of the aortic atherosclerotic lesions in high-fat diet fed ApoE knockout mice. Moreover, peach kernel oil treatment can significantly down regulate the expression of TF protein to inhibit the formation of atherosclerotic plaque. In conclusion, peach kernel oil may be a potential health food to prevent atherosclerosis in cardiovascular diseases.

## 1. Introduction

Atherosclerosis, an advanced chronic inflammatory disease, is the most relevant cause of cardiovascular disorders and cerebrovascular ischemic diseases [1,2]. It involves a series of inflammatory responses within arterial lumen that accumulate plaques, which have a lipid-rich core and fibrous cap. Vascular endothelial cells (VEC) is a layer of cells that forms a barrier between blood stream and vascular smooth muscle, maintaining vascular permeability, secreting vascular active substances, communicating vascular message, which is associated with hemostatic function, anticoagulant, and antithrombotic functions [3]. In vascular homeostasis, the endothelium regulates the vascular dilation and contraction through inhibiting or promoting the migration and proliferation of a layer of muscle cells [4]. Therefore, endothelial dysfunction may induce atherosclerosis development, and tumor necrosis factor α (TNF-α) serves as an inflammatory mediator that can induce endothelial cells to express tissue factor (TF), activating the coagulation reaction, reducing the anti-thrombotic function of endothelial cells, which is causatively involved in the formation and development of atherosclerosis [5,6].

Tissue factor (TF) is the onset of exogenous coagulation factor, playing an important role in the formation of atherosclerosis [7,8,9,10]. After the atheromatous plaque rupture, plaque and TF in VEC entered the bloodstream, starting the exogenous coagulation process, leading to atherosclerosis [9]. Overexpression of TF in rat arterial neointima can model thrombosis and progression of advanced atherosclerosis [11]. Due to the regulatory function of endothelial cells, the TF associated with vasoconstriction or vasodilation have been considered as the key for atherosclerosis development [12,13]. Numerous therapies focus on lipid-lowing property to reverse the atherosclerosis. It has been demonstrated that peach kernel showed therapeutic function on cardiovascular diseases [14]. Peach kernel is one of the nine plant ingredients used in a cocktail for cardiovascular disease [15]. In previous study done by Rahma and El-Aal, the major component of kernels of peach fruits contain 54.5% and 27.5% oil and protein, respectively, but ash and total carbohydrates were quite low [15]. The fat-soluble components in peach kernel oil’s accounted for about 50% of the dry weight of the peach kernel [16], which may have clinical benefits in Chinese herbal remedies [17]. In addition, the dietary administration of apricot kernel oil in rat can causes improvement in liver antioxidant status of rats in comparison to sunflower oil which is a commonly consumed vegetable oil [18]. The administration of apricot kernel oil in rats can significantly ameliorate inflammatory response [19]. This observation promotes us to test the potential anti-atherosclerosis function of peach kernel oil in this study.

Apolipoprotein E (ApoE) plays an important role to maintain the normal levels of cholesterol and triglyceride in the serum by transporting fat (lipids) in the bloodstream from one tissue to another [20]. Mice deficient for ApoE function showed hypercholesterolemia, increased VLDL, decreased HDL, displayed spontaneous plaques formation aged at 3 months-old, and this plaques/lesions formation can be greatly accelerated after feeding with high-fat diet [21]. After applying the peach kernel oil, HUVEC endothelial cells showed increase averting the negative effect of the TF expression induced by TNF-α. In addition, we investigated the effects of peach kernel oil’s on ApoE knockout mice, and observed decreased blood lipid levels, reduced size of the atherosclerotic lesions, and downregulated expression of the TF within the artery atheromatous plaques, which lent support for the exploring further the potential clinical application of the peach kernel oil in treating cardiovascular diseases.

## 2. Results

### 2.1. The Fatty Acid Composition of Peach Kernel Oil

**Figure 1:**
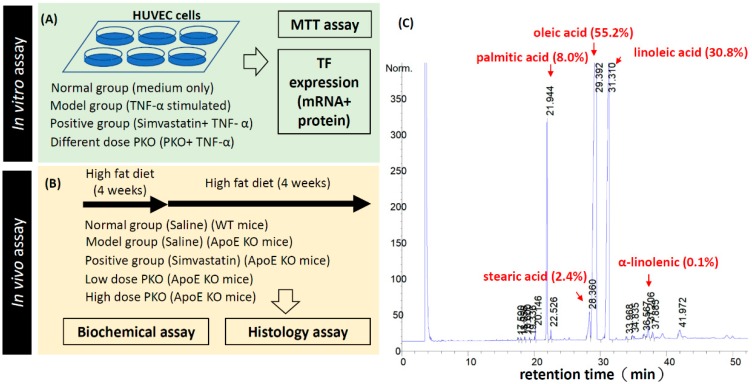
Experimental design, workflow and chemicals used in this study. (**A**) Peach kernel oil on human umbilical vein endothelial cells (HUVEC) endothelial cells viability; (**B**) Peach kernel oil on reducing atherosclerosis in ApoE knockout (KO) mice; (**C**) GC-MS analysis of the major components of peach kernel oil. PKO stands for peach kernel oil.

The experimental procedure and workflow to test the potential anti-atherosclerosis function of the peach kernel oil in vitro and in vivo was summarized in Figure 1A,B. Gas chromatography–mass spectrometry (GC-MS) was used to analyze the fatty acid composition of the peach kernel oil. The map for the total ion chromatograms of methyl-esterized peach kernel oil was shown in Figure 1C. Unsaturated fatty acid content in peach kernel oil accounted for 86% of the total fatty acid content. The main unsaturated fatty acid was oleic acid (55.2%) and linoleic acid (30.8%); the saturated fatty acids mainly were palmitic acid (7.97%), stearic acid (2.37%), and α-linolenic acid (0.11%) (Figure 1C). The lipid composition of peach kernel oil tested in this study is similar to previous report showing oleic acid and linoleic acid are two major unsaturated fatty acid in peach kernel oil [15].

### 2.2. Peach Kernel Oil Affects HUVEC Viability and Inhibits TNF-α-Induced TF mRNA Expression

**Figure 2:**
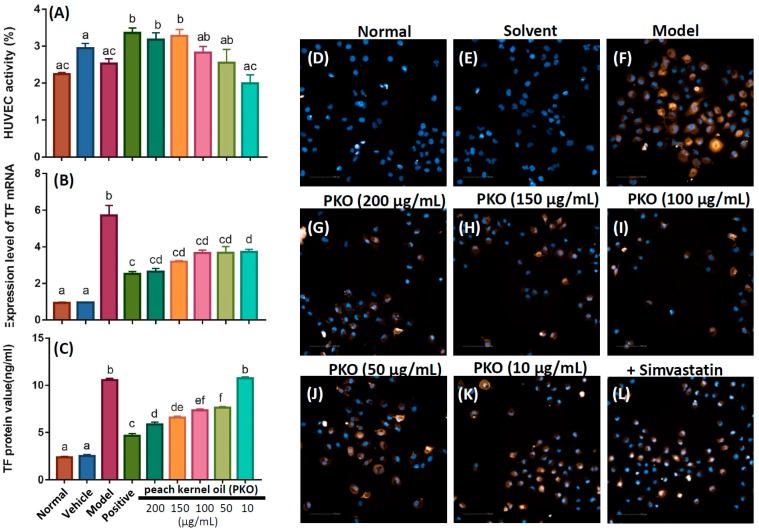
Cell viability, TF expression at mRNA and protein levels in HUVEC after treated with different concentration of peach kernel oil. No chemicals were added in the normal group. As a vehicle control, DMSO was added for evaluating the solvent effect. For the model group, TNF-α was added to the culture medium to stimulate TF protein expression. For the positive group, simvastatin was added to HUVEC to suppress the TF protein induction by TNF-α stimulation. After pretreating HUVEC with simvastatin or peach kernel oil, TNF-α protein was added to HUVEC culture medium to induce TF protein expression. (**A**) Cell viability of HUVEC after treated with different concentrations of peach kernel oil. The TF expression at mRNA (**B**) or protein (**C**) level in HUVEC after treated with different concentrations of peach kernel oil. (**D**–**L**) The expression of TF protein in HUVEC after treated with peach kernel oil detected by immunohistochemistry (yellow color). The cell nuclei were counterstained with DAPI. PKO stands for peach kernel oil. Different labels above columns in (**A**–**C**) indicate a significant difference tested by One-way ANOVA between different experimental groups with *p* < 0.01.

The effect of peach kernel oil on cell viability of HUVEC was tested by MTT assay. We did not add any chemicals in the normal group. As a vehicle control, DMSO was added for evaluating the solvent effect. For the model group, TNF-α was added to the culture medium to stimulate TF protein expression. For the positive group, simvastatin was added to HUVEC to suppress the TF protein induction by TNF-α stimulation. We found that HUVEC viability significantly increased in the positive group (+simvastatin) and in peach kernel oil treated group (Figure 2A). In the peach kernel oil-treated groups, we found the HUVEC viability and the amount of exogenous peach kernel oil added display a positive dose-dependent relationship. It is intriguing to find the HUVEC viability can reach similar level to the positive group (+simvastatin) after receiving a very low dose peach kernel oil treatment (0.05 μg/L).

In the positive control group (+simvastatin) and peach kernel oil treated group (from 0.01 to 0.2 μg/mL), chemicals were added to HUVEC culture medium as a pretreatment. Then, TNF-α protein was added to the culture medium induce TF mRNA expression. As a vehicle group, DMSO was added to evaluate the solvent effect. The result showed that the expression of TF mRNA in the model group (+TNF-α protein) was significantly elevated. However, when either simvastatin (positive group) or different doses of peach kernel oil were applied, the TF mRNA expression levels were significantly decreased, suggesting that peach kernel oil inhibits TF mRNA induction in vitro (Figure 2B). We found that the TF mRNA expression can reach similar level with the positive group (+simvastatin) after receiving a very low dose peach kernel oil treatment (0.05 μg/L). This might be due to different mechanism between simvastatin and peach kernel oil on decreasing TF expression.

### 2.3. Peach Kernel Oil Decreases TNF-α-Induced TF Protein Expression in HUVEC

To provide quantitative data, we applied ELISA to validate TF protein expression in HUVEC. After pretreating HUVEC with simvastatin or peach kernel oil, TNF-α protein was added to HUVEC culture medium to induce TF protein expression. The results showed that the TNF-α could significantly promote TF protein expression as compared to the normal or vehicle group. Simvastatin and different doses of peach kernel oil significantly inhibited TF protein expression in HUVEC (Figure 2C). More peach kernel oil added, more significant lower level of TF protein was detected.

We also performed immnuohistochemistry (IHC) to compare TF protein expression in situ in HUVEC after receiving different treatments. As expected, the results showed that the TNF-α could significantly elevate TF protein expression in HUVEC in the model group (yellow, Figure 2F). The normal or solvent group, on the contrary, TF protein was barely detected (Figure 2D,E). After administration of simvastatin, the cellular level of TF protein expression was reduced (Figure 2L). The administration of peach kernel oil also significantly inhibited the TF protein expression in HUVEC in a dose-dependent manner (Figure 2G–K).

### 2.4. Detection of TC, TG, HDL-C and LDL-C Levels

**Figure 3:**
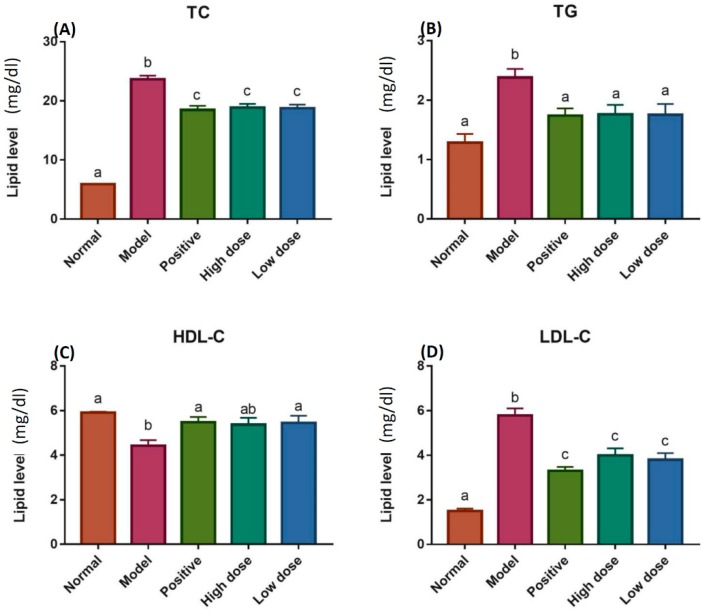
The lipid levels of TC, TG, HDL-C and LDL-C in the serum of ApoE-knockout (KO) mice. (**A**) Total cholesterol (TC), (**B**) Triglyceride (TG), (**C**) High-density lipoprotein cholesterol (HDL-C), and (**D**) Low-density lipoprotein cholesterol (LDL-C) levels in the serum were measured after feeding with high-fat diet for 8 weeks. Different labels above columns in (**A**–**D**) indicate a significant difference tested by One-way ANOVA between different experimental groups with *p* < 0.01.

For the in vivo assay, we fed ApoE knockout (KO) mice with high-fat diet for four weeks and later the potential anti-atherosclerosis effect of peach kernel oil in ApoE KO mice was evaluated (Figure 3). Both normal and model groups of mice were supplement a high-fat feeding for 8 weeks to establish the model of atherosclerosis in wild type and ApoE KO mice. The high-fat diet containing 21% fat, 0.5% cholesterol and 78.5% other basic constitutions. After the high-fat feeding for 4 weeks, mice were subjected 5 mg·kg^−1^·d^−1^ of simvastatin, high dose (2 g·kg^−1^·d^−1^), or low dose (5 g·kg^−1^·d^−1^) of peach kernel oil. The animals were sacrificed and total cholesterol (TC), triglyceride (TG), low-density lipoprotein cholesterol (LDL-C) levels, and the high-density lipoprotein cholesterol (HDL-C) levels in the serum was compared. Compared to the normal group, TC, TG and LDL-C levels were elevated; while the HDL-C level was significant reduced in the model group (ApoE KO mice receiving saline). For positive group (ApoE KO mice receiving simvastatin), we found administration of simvastatin significantly restored the phenotype detected in the model group, showing reduced TC, TG and LDL-C levels and elevated HDL-C level. It is intriguing to find that the administration of peach kernel oil even at dose as low as 2 g·kg^−1^·d^−1^ could generate a similar rescue effect with simvastatin positive group (Figure 3).

### 2.5. Peach Kernel Oil Reduced Aortic Atherosclerosis in ApoE KO Mice

**Figure 4:**
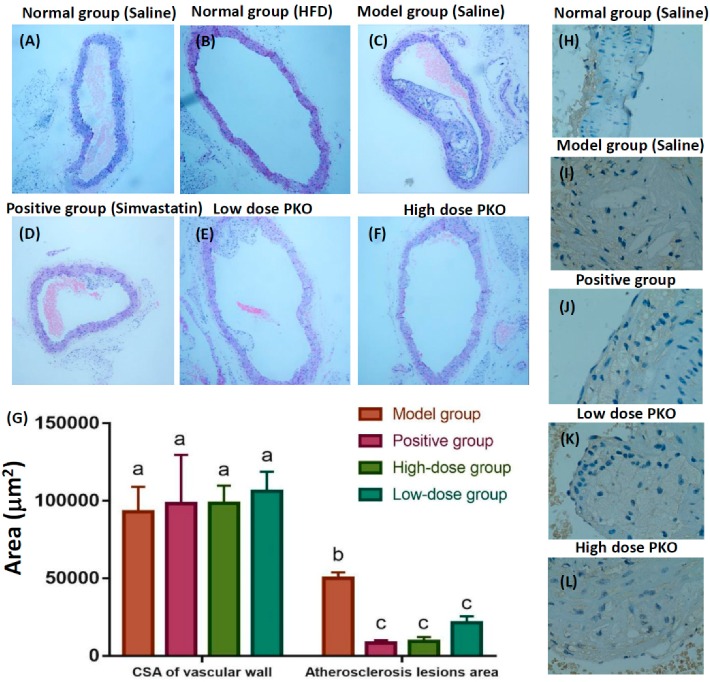
Histology and immunohistochemistry assay of the aorta of ApoE knockout (KO) mice at 400× magnification after treating with peach kernel oil (PKO). (**A**) Normal aorta in normal mice without high-fat diet feeding (normal group); (**B**) Aorta in normal mice fed with high-fat diet feeding for eight weeks (normal group); (**C**) Aorta in ApoE KO mice fed with high-fat feeding for eight weeks (model group); (**D**) Aorta of ApoE KO mice fed with high-fat feeding and simvastatin (positive group); (**E**) Aorta of ApoE KO mice fed with high-fat feeding and high dose peach kernel oil (PKO); (**F**) Aorta of ApoE KO mice fed with high-fat feeding and low dose PKO (**G**) Quantitative results from lumen area (statistics was tested by One-way ANOVA and Tukey-HSD test, *p* < 0.01). Different labels above columns in (**G**) indicate a significant difference tested by One-way ANOVA between different experimental groups with *p* < 0.01. Immunohistochemistry assay showing TF protein expression level in atherosclerotic plaques in (**H**) normal group (**I**) model group (**J**) positive group (**K**) high dose PKO treatment and (**L**) low dose PKO treatment groups.

In the normal group (wild type C57BL/6 strain mice receiving saline), it was seldom to detect aortic atherosclerosis formation after 8 weeks of normal diet (Figure 4A) or high-fat diet feeding (Figure 4B). In the model group (ApoE KO mice fed with high-fat diet for 8 weeks), atherosclerosis lesions, lumen blocking, disorder of smooth muscle, formation and accumulation of many foam cells, large number of inflammatory cells, and atherosclerosis plate-cholesterol clefts were detected (Figure 4C). Simvastatin is currently considered as an effective treatment for decreasing serum lipid level [22,23]. ApoE KO mice treated with simvastatin were used as a positive group for evaluating potential anti-atherosclerosis lesions function of the peach kernel oil (Figure 4D). Mice treated with either high or low dose of peach kernel oil, the degree of aortic atherosclerosis and inflammation were found to be significantly attenuated (Figure 4E,F). The index of aortic atherosclerotic lesions was listed in Table 1. Compared with the normal group, aortic atherosclerotic lesions area of mice in the positive group, high and low dose peach kernel oil groups decreased significantly (Figure 4G), the aortic atherosclerotic lesions area and the aortic atherosclerotic lesions area/cross-sectional area (CSA) of the vascular wall also reduced significantly (Table 1).

### 2.6. Peach Kernel Oil Reduced the TF Protein Expression in Atherosclerotic Plaques

Tissue factor (TF) is the onset of exogenous coagulation factor, playing an important role in the formation of atherosclerosis [7]. Therefore, we measured the TF protein expression within the atherosclerotic plaques in mice by immunohistochemical method to evaluate the potential anti-atherosclerosis effect of peach kernel oil. TF protein expression in aorta vascular tissue of normal mice was at low level and only can be detected in the outer membrane (Figure 4H). Compared with the normal group, the aortic TF expression increased noticeably in model group, while TF widely expressed in the plaque intima fiberous connective tissue (Figure 4I). Compared with model group, the expression of TF protein in positive (+simvastatin) (Figure 4J), high (Figure 4K) and low (Figure 4L) doses of peach kernel oil groups were slightly reduced.

### 2.7. Peach Kernel Oil Reduced the Inflammatory Response In Vitro

**Figure A1:**
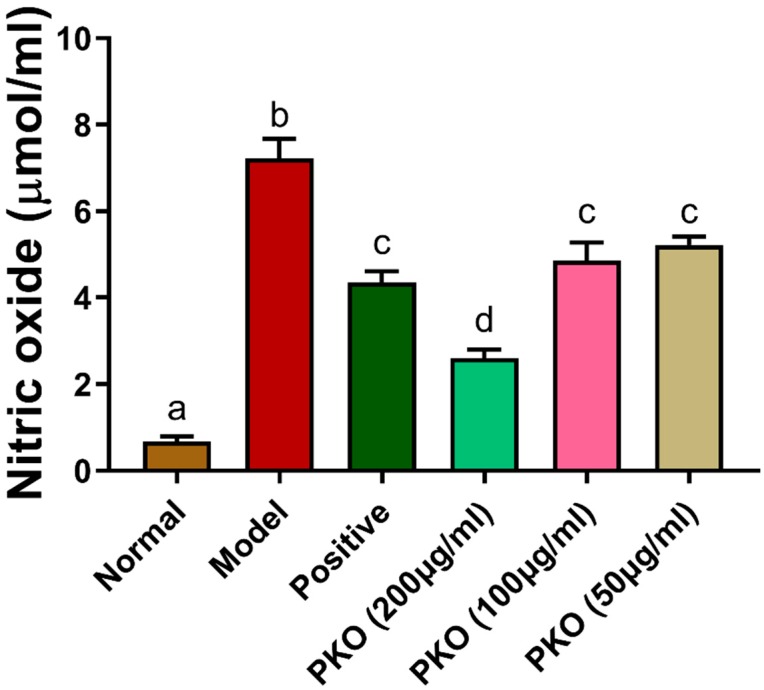
The potential anti-inflammatory function of peach kernel oil on suppressing nitric oxide (NO) release from RAW264.7 macrophage cell lines after challenged with lipopolysaccharide (LPS). Different labels above columns indicate a significant difference tested by One-way ANOVA and Tukey-HSD test with *p* < 0.01. For normal group, there are no chemicals were added. For model group, lipopolysaccharide (LPS) at 1 μg/mL concentration was used to induce inflammation and nitric oxide (NO) release. For positive group, dexamethasone at 1 μg/mL concentration was co-incubated with LPS at 1 μg/mL concentration to attenuate inflammation and nitric oxide (NO) release. For Peach kernel oil (PKO) groups, PKO at different concentration of 200, 100 and 50 μg/mL were co-incubated with LPS at 1 μg/mL concentration to test its potential function on anti-inflammation and suppress NO release.

To explore the potential mechanism for peach kernel oil on anti-atherosclerosis, we performed in vitro assay to test the potential anti-inflammatory function of peach kernel oil on suppressing nitric oxide (NO) release from RAW264.7 macrophage cell lines after challenged with lipopolysaccharide (LPS). For model group, we found the LPS administration can significantly stimulate the inflammation response on elevating NO releasing level. However, this LPS-induced inflammation can be attenuated when dexamethasone (an anti-inflammation drug) was administrated. By co-incubated with peach kernel oil at high dose and LPS, we found the NO level can be significantly reduced to a level similar normal group when peach kernel oil was given at 200 μg/mL, and to a level close to positive control of dexamethasone when peach kernel oil was given at either 100 or 50 μg/mL (Figure A1). Those results clearly demonstrated peach kernel oil display anti-inflammation effect when tested in vitro on RAW264.7 macrophage cell lines.

## 3. Discussion

Atherosclerosis is one of the important risk factors to induce cardiovascular diseases, such as coronary heart disease, cerebral infarction, and myocardial infarction, which has become a serious threat to human health [1,24]. ApoE KO mice is one of the common animal models that has been used for atherosclerosis research in vivo [6,25]. The mice that were lack of the ApoE activity spontaneously led to atherosclerosis due to high total cholesterol levels [26]. In our study, we observed that the ApoE KO mice fed with high-fat diet for 8 weeks, showed a serious hyperlipidemia at 16 weeks of age. The aortic root formed atherosclerosis, which confirmed atherosclerosis model was successfully established. Compared with the model group (treated with simvastatin), the aortic atherosclerotic area in different doses of peach kernel oil groups were reduced, which illustrated that peach kernel oil indeed can inhibit the development of atherosclerosis.

Alterations of endothelial cells and the vasculature play important roles in the pathogenesis of human diseases. Endothelial cells have the key function on maintaining functional capillaries [27,28]. The endothelium is directly involved in peripheral vascular disease, stroke and heart disease. Endothelial cells form the luminal vascular surface and thus play crucial role on regulating coagulation. Endothelial cells control the clotting system by regulating the expression of binding sites for anticoagulant and procoagulant factors on the cell surface. In the quiescent state, endothelial cells maintain blood fluidity by promoting the activity of numerous anticoagulant pathways, including the protein C pathway. After activation, the balance of endothelial properties can favor the clot formation through the coordinated induction of procoagulant and suppression of anticoagulant mechanisms. Tumor necrosis factor suppresses the formation of throbomodulin, an endothelial anticoagulant cofactor, and induces the expression of tissue factor (TF), which is a procoagulant cofactor [29]. Under the condition of damaged endothelial cells, a high level of TF was released and promoted thrombosis. TF is the transmembrane singly linked glycoprotein and composed by 263 amino acid residues [30]. Blood cells and endothelial cells were directly contacted with the circulation, which do not express tissue factor in general condition. Many stimulating factors, such as bacterial endotoxin, tumor necrosis factor, and type oxidized low density lipoprotein (ox-LDL) attack vascular endothelial cells and monocytes to express TF [31,32]. Excessive expression of TF was closely related to the thrombosis of many diseases, such as sepsis, cancer and atherosclerosis [33]. TNF-α is a kind of inflammatory cytokines that affects the anti-coagulant property of vascular endothelial cells. It was secreted by mononuclear macrophages and effected on vascular endothelial cells by the damage [6]. It also induced proliferation of mononuclear cells, endothelial adhesion, and inward transfer subcutaneous clearance, to form the smooth muscle cells associated with sex foam cells and macrophage foam cells, which showed impacts on the stability of atherosclerotic plaque [34]. Previous studies demonstrated that TNF-α damaged human umbilical vein endothelial cells (HUVEC) and released inflammatory factor TF [35].

**Figure 5:**
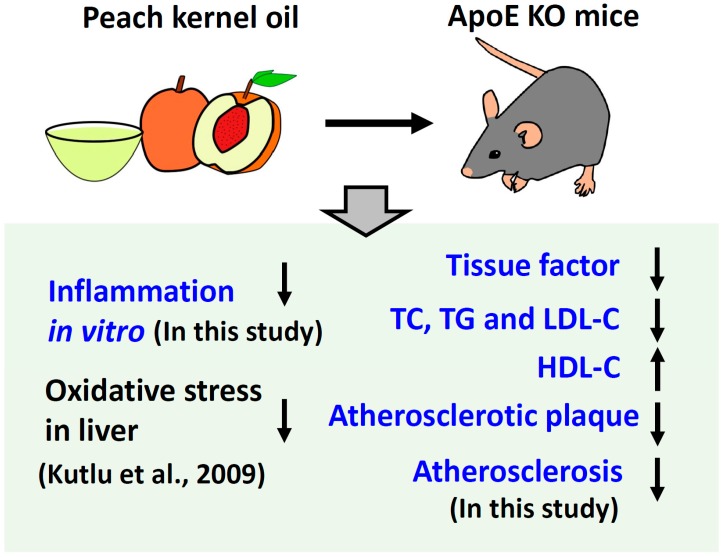
Schematic representation of the obtained results and the protective effects of peach kernel oil to reduce Atherosclerosis in ApoE knockout Mice. The results obtained from this study are highlighted in blue color. The result obtained from previous study [18] is highlighted in black color.

Peach kernel promotes blood circulation and removes blood stasis [36]. It has the significant clinical efficacy that significantly improved the blood rheology and microcirculation in the animal model of stasis heat exchange and haemorrheological nature blood stasis. Peach kernel oil also can improve microcirculation of normal mice and hemorheology index in rats [37]. Peach kernel oil is thought to be the main active constituents of peach kernel to promote blood circulation and to remove blood plaques. In this study, the effect of peach kernel oil on atherosclerosis were demonstrated, both by in vivo and in vitro experiments. Results showed that peach kernel oil not only reversed the abnormal blood lipid levels, but also reduced the degree and area of the aortic atherosclerotic lesions in the ApoE KO mice. It also reduced the levels of TF protein expression in artery atheromatous plaques, which suggested an effect of deceleration of the atherosclerosis process (summarized in Figure 5). Peach kernel oil may affect the activation of blood coagulation system and maintain the dynamic balance in endogenous vascular active substances, thereby improve the thrombotic diseases such as atherosclerosis. By collection of in vitro evidence in this study and in vivo evidence in previous study [18], we proposed the anti-atherosclerosis function of peach kernel oil might related to its anti-inflammatory (Figure A1) and anti-oxidative capacity. In conclusion, by combining much in vitro and in vivo evidence, we provided strong evidence showing peach kernel oil showed great activity on down-regulating the expression of TF protein to inhibit the formation of atherosclerotic plaque, which may have potential to develop into health food to prevent atherosclerosis in cardiovascular diseases in the future.

## 4. Materials and Methods

### 4.1. Extraction of Peach Kernel Oil

Peach kernel (batch number 13110814) was originally collected from Hebei, China and purchased from People Big Pharmacy Corporation in Guangxi, China. Twenty grams of peach kernel was extracted by 130 mL petroleum ether extraction solvent at 65 °C for 2.5 h. The extract was refluxed in water bath pot, and filtered. The organic solvent was removed in water bath at 37 °C. Oil rate was weighed and calculated. Extraction rate was calculated as: actual amount of oil extraction (g)/quality of raw material (g) × 100%.

### 4.2. Analysis of Fatty Acid Composition

After peach kernel oil undergoing methyl-ester process, the fatty acid composition of peach kernel oil was analyzed by GC-MS chromatograph (Agilent 6890N, Santa Clara, CA, USA). GC-MS conditions: 30 m × 0.25 mm × 0.25 μm capillary column with a flow rate of carrier gas at 0.7 mL/min. The split ratio was 50:1, and vaporizing chamber temperature was 240 °C. The column temperature was at 180 °C with 1 μL sample quantity. Needing EI ion source temperature was at 230 °C. The electron energy was at 70 ev. The electronic energy multiplier voltage was at 0.9 kV, with 40–600 amu scanning range.

### 4.3. Cell Culture, Drug Treatment and Grouping

HUVEC, purchased from Shanghai medical college, were grown in DMEM (containing 10% fetal bovine serum, 100 u/mL penicillin and streptomycin) at 37 °C with 5% CO_2_. Peach kernel oil was dissolved by protein adsorption method, which dissolved peach kernel oil in anhydrous ethanol to reached the concentration of 20 μg/mL. Peach kernel oil was preheated at 55 °C and added into DMEM with 1% bovine serum albumin (BSA). Mixture was dissolved by the oscillation at 55 °C water bath before experiment. HUVEC at the density of 5 × 10^6^/mL were inoculated onto 6-well petri dish. Normal group: cells in serum-free 20 μL DMEM medium without drugs. Normal vehicle group: cells in 20 μL 1% anhydrous ethanol, 1% BSA, serum-free 1% DMEM medium without drugs. Model group: cells in TNF-α (final concentration at 20 ng/mL). Positive group: simvastatin (final concentration of 5 μmol/L) was added after 18 h incubation, 20 μL TNF-α (final concentration for 10 ng/mL). Each dose Peach kernel oil groups: different concentrations of peach kernel oil (0.01, 0.05, 0.1, 0.15, 0.2 μg/mL) were added after the incubation time for 18 h, then TNF-α (final concentration at 10 ng/mL) was added and incubated for 6 h.

### 4.4. MTT Assay

Cells were incubated in 10 μL MTT reagent (5 mg/mL) for 4 h at 37 °C and 5% CO_2_. Removed the MTT reagent and washed before adding 100 μL DMSO to each well for 10 min to dissolve formed crystal. Spectrophotometer was used to determine absorbance at 570 nm. The average absorbance and the cell vitality were calculated as follow: cell viability = (OD value of dosing group/OD value of normal group) × 100%. The OD value of dosing group and normal group through the blank group.

### 4.5. Anti-Inflammatory Effect of Peach Kernel Oil

RAW264.7 macrophage cell lines were incubated in Dulbecco’s Modified Eagle Medium (DMEM) medium with 1% BSA at 37 °C and 5% CO_2_. For normal group, there are no chemicals were added. For model group, lipopolysaccharide (LPS) at 1 μg/mL concentration was used to induce inflammation and nitric oxide (NO) release. For positive group, dexamethasone at 1 μg/mL concentration was co-incubated with LPS at 1 μg/mL concentration to attenuate inflammation and nitric oxide (NO) release. For Peach kernel oil (PKO) groups, PKO at different concentration of 200, 100 and 50 μg/mL were co-incubated with LPS at 1 μg/mL concentration to test its potential function on anti-inflammation and suppress NO release.

### 4.6. Quantitative Realtime-PCR (qRT-PCR)

Reverse transcription was performed to synthesis cDNA by using commercial kit (Dalian Treasure Biological Engineering). PCR primers was purchased from Beijing liuhe genomics technology as followed: TF forward 5′-CTGAGCCCTTCCTTCTCAGC-3′, the reverse 5′-GATCACGCAGACCCTTTTAGA-3′; ACTB forward 5′-CAGGCACCAGGGCGTGAT-3′, the reversed 5′-TAGCAACGTACATGGCTGGG-3′. The relative expression of TF mRNA was calculated by the 2^−∆∆*C*t^ method [38].

### 4.7. Preparation of the Mouse Model of Atherosclerosis

ApoE knockout and normal mice (C57BL/6 strain) at 8 weeks of age were purchased from Health Science Center in Peking University. Thirty-two male ApoE knockout mice (C57BL/6 strain) aged at 8 weeks old were randomly divided into four model groups: model, positive, high dose and low dose peach kernel oil group, while 8 wild type male mice as the normal group. Mouse were given a high-fat feeding for 8 weeks to establish the model of atherosclerosis in wild type and ApoE knockout mice. The high-fat diet containing 21% fat, 0.5% cholesterol and 78.5% other basic constitutions. After the high-fat feeding for 4 weeks, mice were fed with 5 mg·kg^−1^·d^−1^ of simvastatin, high dose (2 g·kg^−1^·d^−1^), or low dose (5 g·kg^−1^·d^−1^) of peach kernel oil. All experimental protocols and procedures involving mice were approved by the Committee for Animal Experimentation of Guangxi University of Chinese Medicine (ethical permission code SCXK-2014-0002 and permission date 24 December 2013). All experiments were performed in accordance with the guidelines for laboratory animals.

### 4.8. Sacrifice and Processing of the Specimens of Mice

Mice were under fasting for more than 12 h before death. 0.2–0.3 mL of 20% urethane saline solution injection was used to anesthesia mice. Saline and 4% paraformaldehyde in PBS were used to perfuse and fix aortic for 15 min. Completely separation the heart and the aorta by breaking out the heart and bifurcation form the aortic root to iliac artery. After taking 1.0 cm the aorta in aortic root of all mice, 4% paraformaldehyde was used to fixation, then performed the paraffin sectioning.

### 4.9. Blood Lipid Index

The total cholesterol (TC), triglyceride (TG), high-density lipoprotein cholesterol (HDL-C) and low-density lipoprotein cholesterol (LDL-C) level in serum were determined by the commercial kits (Chang Chun HuiLi Biotech Co., LTD).

### 4.10. Histology

Specimen from aorta was fixed in 4% paraformaldehyde and conventionally embedded in paraffin. Sample was cut into 5 μm in each section and H&E staining was applied. Image analysis was done by Image Pro Plus 6.0 software (http://www.mediacy.com/imageproplus). The size of mouse artery atheromatous plaque was measured by the scale on blood count plate. The diameter of blood vessel wall was calculated from outside boundary of artery elastic layer to the elastic layer according to previous published method [39]. Paraffin slides were de-wax and then performed immunohistochemical staining with rabbit anti-TF monoclonal antibodies (Abcam) at 1:100 dilution. After immunohistochemical staining, TF protein staining in the arterial wall and plaques, was developed by DAB Peroxidase Substrate Kit.

### 4.11. Detection of TF Protein Content by ELISA

The endothelial cells were washed with PBS three times before adding 15 nM octyl-B-d-glucopyranoside to lyse cells at 37 °C for 15 min. Cell extract was collected and incubated with 50 μL standard solution in each well at room temperature for 2 h. The TF protein content was later quantified by TF ELISA kit (ET1002-1, AssayPro) according to the manufacture’s instruction at wavelength of 450 nm.

### 4.12. Statistical Analysis

Graphpad prism software (https://www.graphpad.com/scientific-software/prism/) was used for statistical processing and the results were expressed as Average ± standard error of mean ($\bar{X}$ ± SEM). Data were analyzed by Student’s two-tailed-*t*-test for comparison between two groups or one-way ANOVA for multiple group comparison. *p* value of less than 0.05 was considered statistically significant.

## Figures and Tables

**Table 1:** The comparison of aortic atherosclerotic lesions area in mice ($\bar{X}$ ± SD, *n* = 8).

Grouping	Atherosclerosis Lesions Area (μm^2^)	Cross-Sectional Area of Vascular Wall (μm^2^)	Atherosclerosis Lesions Area/Cross-Sectional Area of Vascular Wall
Model group	50,176 ± 5348 ^a^	93,118 ± 22,508 ^a^	0.547 ± 0.074 ^a^
Positive group	8298 ± 2629 ^b^	98,249 ± 44,394 ^a^	0.087 ± 0.013 ^b^
High-dose PKO group	9396 ± 4028 ^b^	98,592 ± 15,878 ^a^	0.093 ± 0.025 ^b^
Low-dose PKO group	21,418 ± 5807 ^b^	106,322 ± 17,706 ^a^	0.199 ± 0.021 ^b^

PKO stands for peach kernel oil; Different labels above data indicate a significant difference tested by One-way ANOVA between different experimental groups with *p* < 0.01.
